# Fugitive Chemicals and Environmental Justice: A Model for Environmental Monitoring Following Climate-Related Disasters

**DOI:** 10.1089/env.2017.0044

**Published:** 2018-06-01

**Authors:** Jaime Madrigano, Juan Camilo Osorio, Eddie Bautista, Ryan Chavez, Christine F. Chaisson, Erika Meza, Regina A. Shih, Ramya Chari

**Keywords:** environmental justice, hurricane, fugitive chemicals, environmental monitoring

## Abstract

The combination of population growth in areas of mixed (residential, commercial, and industrial) land use along U.S. waterfronts and the increasing frequency of devastating hurricanes and storm surges has led to community fears of widespread toxic chemical contamination resulting from accidental industrial or small business releases, particularly in the aftermath of an extreme weather event, such as a hurricane. Industrial waterfront communities, which are frequently environmental justice communities, contain numerous toxic chemical sources located in close proximity to residential housing, schools, daycare centers, playgrounds, and healthcare centers. Despite the longstanding concerns of community activists and researchers about the potential for “fugitive” chemicals to be released into floodwaters, there has been little coordinated research or action to develop environmental monitoring programs for disaster-affected communities. In the aftermath of Superstorm Sandy, a community-academic partnership was formed between the New York City Environmental Justice Alliance, UPROSE, The LifeLine Group, and the RAND Corporation. The collaboration, known as Grassroots Research to Action in Sunset Park (GRASP) has focused on identifying possible sources of chemical contamination, modeling the potential for chemical release into community areas and resulting exposure risks, and proactively developing actions for mitigating or preventing adverse community impacts. Through our ongoing work, we have identified barriers and drivers for community-based environmental monitoring, and in doing so, we have developed a framework to overcome challenges. In this article, we describe this framework, which can be used by waterfront communities bracing to deal with the effects of future devastating weather disasters.

## Introduction

Hurricane Harvey made landfall on August 25, 2017 as a Category 4 hurricane. In its aftermath, concerns about the undefined “toxic soup” of chemicals and pathogens contained in its floodwaters dominated the headlines.^1^ Unfortunately, this is not the first (nor likely to be the last) incident where floodwaters inundate a waterfront community, dislodge chemicals and other contaminants and turn them “fugitive.”

As the amount of people living along the coast continues to increase across the nation,^2^ the release of fugitive chemicals may result in large public health impacts. While supportive of urban development in waterfront areas,^3^ the increase in mixed use communities can lead to concentrated areas of pollution-intensive activity taking place near public spaces and residential locations. Large industrial facilities are often subject to regulations or zoning performance standards that minimize public exposure to dangerous contaminants, but these may be inadequate. For example, in New York City (NYC), these standards, established in 1961, are out of date.^4^ In addition, small industrial businesses oftentimes lack the resources necessary to implement safe chemical storage and disposal practices. Furthermore, many of the protections that are in place are likely to fail during a devastating hurricane because, in many cases, waterfront development has not accounted for climate change projections. As we have seen again and again, with Katrina, Sandy, Harvey, Maria, and numerous other extreme weather events, floodwater inundation can cause chemicals to become fugitive, resulting in uncharted chemical exposure with unknown short-term or durable consequences to human health.

The threats posed by fugitive chemicals are not distributed equally across waterfront areas. A documented history of links between land use and zoning and disproportionate environmental burdens has demonstrated that low-income or communities of color are adjacent to or mixed into areas that have manufacturing and industrial uses.^5^ This is no different in waterfront communities, where ∼12% of the population in the U.S. coastal floodplain lives in poverty.^6^ Disproportionate exposure to toxic chemicals by low-income communities and communities of color occurs in many waterfront communities across the country, including Houston and New York.^7,8,9^

As documented in the aftermath of Hurricane Katrina, community activists and researchers have been concerned about the potential for fugitive chemicals to be released into floodwaters for some time.^10,11,12^ In NYC, small community organizations are leading efforts to publicize and communicate the dangers of fugitive chemicals and spearheading campaigns to create policy change.

In 2010, the New York City Environmental Justice Alliance (NYC-EJA) launched the Waterfront Justice Project. As part of this initiative, NYC-EJA documented that six of NYC's seven Significant Maritime Industrial Areas (SMIAs, zones designed to encourage clustering of heavy industry and infrastructure) are environmental justice communities *and* are in storm surge zones. Through the Waterfront Justice Project, NYC-EJA has conducted research and advocacy to draw political attention to the issues of community vulnerability, and disaster risk reduction, including fugitive chemical releases.^13^ This work has resulted in some notable impacts, including ensuring that NYC's Waterfront Revitalization Program considers climate change impacts and mandate vulnerability assessments for new industrial businesses.

In addition, community members in Sunset Park, Brooklyn, NY, the largest SMIA, came together after Superstorm Sandy and were determined to build resilience ahead of the next storm event. Under the leadership of community group, UPROSE, the Climate Justice Center was formed, which is building the capacity of Sunset Park's indigenous leaders and local businesses to respond to future severe weather events.

The work of local grassroots organizations such as NYC-EJA and UPROSE are critical to building the capacity of environmental justice communities to effect change and reduce the threat of fugitive chemicals after a storm event. However, a major barrier to action on this front is the lack of fugitive chemical measurement. In the wake of Superstorm Sandy, residents of Sunset Park feared widespread toxic contamination; yet, no community-focused environmental monitoring was ever conducted to address their concerns. While investigations by the Department of Environmental Protection did not indicate the presence of spilled chemicals on facility sites, this may have been because the high volume of water had already washed them away.^14^ It is also possible that soil, structures, and debris had adsorbed the chemicals.

Community concern over hazardous chemical releases from industrial sites was documented in the Sandy Regional Assembly's Recovery Agenda, a report issued by an association of over 40 grassroots organizations after the storm, which called for the mitigation of industrial waterfront threats and the identification of risks to public health associated with potential exposures to hazardous substances and toxic chemicals.^15^ Furthermore, concerns about the lack of and need for community-focused environmental monitoring were documented by UPROSE, NYC-EJA, and others in a National Environmental Justice Advisory Council (NEJAC) report to EPA^16^ and by the NYC Panel on Climate Change.^17^ Without measurement, there is no way to document the magnitude and scope of the problem and to understand where resources and interventions are most needed. Thus, community fears remain unaddressed and can be channeled into long-term worry about unknown exposures over time.^18^ Disaster environmental monitoring would benefit communities greatly by increasing community members' knowledge on the risks they are exposed to and ways to protect themselves as well as providing evidence to support advocacy for policy action.

In the aftermath of Superstorm Sandy, the Grassroots Research to Action in Sunset Park (GRASP) partnership was formed (https://www.rand.org/jie/infrastructure-resilience-environment/projects/grasp.html). The GRASP collaboration consists of two community-based organizations (CBOs)—NYC-EJA and UPROSE—and two research organizations—The LifeLine Group and the RAND Corporation. GRASP follows the principles for effective community-based participatory action research (CBPAR). A focus of the GRASP collaboration has been to identify possible sources of chemical contamination in Sunset Park, model the potential for chemical release into community areas and resulting exposure risks, and proactively develop actions for mitigating or preventing adverse community impacts. Through this work, we have faced many obstacles, including a lack of quality data available for both hazard and exposure characterization.

This dearth of information is a barrier to addressing concerns and taking protective action. Even when groups can mobilize quickly to conduct environmental monitoring, community residents may still lack information and an understanding of the problem. For example, in Houston, a team from Baylor Medical College and Rice University, working with the Houston health department and funded by the New York Times, conducted environmental monitoring, but reports indicate that residents remained concerned about the lack of information available to them and the uncertainty of their health risks.^19^ For these reasons, we believe that it is imperative for waterfront communities to develop a community-based environmental monitoring program in advance of severe weather events. Through our ongoing work in Sunset Park, we have identified barriers and drivers for community-based environmental monitoring and, in doing so, have developed a framework to overcome challenges. In this article, we describe this framework, which can be used by other waterfront communities bracing to deal with the effects of future devastating weather disasters.

## Discussion

Our framework for community-based environmental monitoring consists of six steps. We describe each of these steps in further detail below, why they are important, examples of our implementation in Sunset Park, Brooklyn, applicability to recent disasters, and recommendations for waterfront communities across the United States. Who will spearhead an environmental monitoring effort will vary for each community. In our case, our community-based collaboration has been leading this charge, but in other cases, this may naturally fall to other leaders. Nonetheless, we believe it is worthwhile for all communities to form a broad-based collaboration to ensure stakeholder support for the monitoring program, and therefore, we consider this as the first step in the framework.

### Step 1: form a community-based collaboration

Environmental justice places community agency at the center of its theory of change.^20,21^ Therefore, solutions to environmental justice problems must apply a community-based approach. CBPAR can be a useful lens for thinking about environmental monitoring for disasters because projects are meant to produce results that help communities decide how to act on the problems they are facing.^22^ The formation of a collaborative between a diverse group of actors—including community leaders, members, scientists, policymakers, and so on—is a foundational step for CBPAR work. A central premise is that scientists and community partners work together in *all* phases of the research.^23,24^

Such partnerships require trust and mutual accountability and, therefore, can take time and consistent effort and communication to build and sustain. However, we maintain that this continued effort is crucial for disaster preparedness planning, where community involvement is a key factor in developing sustainable and effective strategies. The more a community can contribute and be involved in the decision-making process with respect to their needs and what they consider feasible practices, the better informed they will be and the more inclined they will be to follow procedures that they helped develop. In some communities, local emergency planning committees (LEPCs) may have plans in place to partially or fully address community concerns. This was not the case in Sunset Park, where such efforts have been led by grassroots CBOs. However, wherever feasible, LEPCs should be approached by the community-based collaborative to synergize efforts and identify specific gaps that can be filled by the community-based effort to avoid redundancy. Throughout our work, our collaborative has engaged with a community stakeholder group (CSG). The CSG, composed of NYC and Sunset Park business and community leaders, provides guidance and oversight as well as thoughtful feedback on how to disseminate our findings in a way that creates awareness and empowerment, but does not cause unnecessary stress or alarm. The goal of this collaboration is to lessen the fear around toxic chemicals that has existed during previous disasters,^25,26,27^ and instead, empower the community to take action on environmental monitoring.

### Step 2: identify shared goals for monitoring

An important piece of the process is to ensure that any monitoring data collected will be useful for a multitude of objectives identified by stakeholders. Ultimate use of the data will directly inform the type of data needed and how it is collected; therefore, objectives for monitoring should be identified early in the process. In our work in Sunset Park, we identified several potential uses of environmental monitoring data. While communities may have different objectives than those listed here, we use them as examples to demonstrate how they can inform monitoring design considerations.

A priority concern among our collaborative was to ensure that effective protection strategies for response and recovery workers and community volunteers could be used in future events. Therefore, one goal of monitoring is to identify the physical properties (e.g., volatility and permeability) of contaminants to facilitate appropriate recommendations for personal protective equipment (PPE). Environmental monitoring from one event can be used to inform protection strategies for future events (assuming no changes to major sources of contamination), or real-time monitoring can be used to inform decision-making on PPE in the field while response and recovery operations are taking place (e.g., alerting recovery workers or volunteers to avoid specific areas or enter only with adequate protective coverage).

A second priority for our community collaborative is to use monitoring data to provide evidence for future action (e.g., site cleanup and policymaking). For this objective, data will need to adhere to a basic set of quality control criteria, which can be established by consulting with scientific experts or regulatory agencies. Another possible objective for environmental monitoring, as demonstrated during Hurricane Harvey, is to alert residents to take precautions when returning to their homes. Each of these objectives may have different requirements for data quality control and turnaround time. Early identification of objectives will allow monitoring procedures for multiple objectives to be developed along parallel tracks, if necessary.

### Step 3: establish an implementation protocol

To implement environmental monitoring in a timely manner when a disaster strikes, procedures must be set up in advance. As part of this planning process, it is important to establish partnerships and obtain buy-in from major stakeholders, including government agencies, academic partners, private companies, and community organizations. Each of these organizations could be critical to the approval, funding, or “boots on the ground” aspect of the monitoring program. This may include the participation and training of citizen scientists, which can increase transparency and credibility of the results from the community perspective.

In addition, establishing baseline conditions, through the performance of predisaster monitoring, is a way to ensure that monitoring results can be interpreted appropriately and accepted as technically valid and scientifically relevant. The implementation protocol can include procedures for performing baseline and postdisaster monitoring, including when to perform it, prioritizing chemical contaminants, and selecting sampling locations. These substantive recommendations for the implementation protocol are further described in steps 4 and 5.

### Step 4: prioritize chemicals of concern

Knowing what contaminants to test for can be one of the biggest challenges a community faces in the chaotic aftermath of a disaster, since a wide variety of chemicals can be present in a mixed-use community and very little information about these hazardous substances may be available. We recommend prioritizing chemicals of concern according to three considerations: (1) quantity of chemical likely to be present in community, (2) potential for chemical to become fugitive during a severe storm or flood, and (3) the toxicity of a fugitive chemical under “realistic” poststorm exposure scenarios. This prioritization scheme requires knowing which chemicals are present in large quantities in the community, and this can be facilitated through the development of a community chemical inventory.^28^

Details of the community chemical inventory we created for Sunset Park, Brooklyn will be described in a forthcoming publication; however, an overview of this procedure is described in this article. In brief, using publicly available data (see Table 1, e.g., of documentation that may be used), large and small chemical source points can be identified in the community and then chemical characteristics extracted (e.g., amount of chemical stored, type of container stored in, and hazardous properties) into a central database. After identifying and prioritizing chemicals likely to be in the community in large quantities, criteria for elimination from consideration for measurement can be developed. For example, available information may indicate that some chemicals have a low probability of release (e.g., stored in tanks), making these chemicals less of an immediate public concern postdisaster. Communities also need to consider chemicals that may exist on industrial sites that are no longer active. As we saw in Hurricane Harvey, 13 of the 41 Superfund sites in Texas were flooded.^29^ Furthermore, other types of sources, such as those found in small chemically intense businesses (e.g., dry cleaners) or household chemicals, may be a priority for certain communities, depending on the distribution of businesses and residential locations.

**Table 1.** Example Reference Sources for Chemical Hazard Assessment

*Reference source*	*Description*
Chemical source points	
Clean Air Act	Location of major dischargers of air pollutants (stationary sources)
CERCLA	Superfund and brownfields sites
Clean Water Act	Major point sources of water pollution
EPCRA	Facilities reporting to the TRI and CRTK laws/programs
SSTS Pesticides Program	Pesticide producing facilities
Resource Conservation and Recovery Act	Hazardous waste treatment, storage, and disposal, and hazardous substance large quantity generators
City/County Planning Departments	Datasets depicting the location, land use, and zoning information by tax block
Onsite chemicals and characteristics	
EPA TRI Form R	Form R reports the maximum amount stored onsite annually for locations required to report to EPA
EPCRA Tier II Emergency and Hazardous Chemical Inventory Forms	Community right-to-know inventory forms provided by businesses upon request
State Departments of Environmental Protection	Records for waste transfer, wastewater discharge, and environmental remediation sites

CERCLA, Comprehensive Environmental Response, Compensation, and Liability Act; CRTK, Community Right-to-Know; EPCRA, Emergency Planning and Community Right-to-Know Act; EPA, Environmental Protection Agency; SSTS, Section Seven Tracking System; TRI, Toxics Release Inventory.

Finally, communities can prioritize chemicals that would be toxic under “realistic” exposure scenarios. For example, in our work focusing on recovery workers and volunteers, we excluded elemental metals that would not be adsorbed and chemicals that were not associated with adverse health effects that could be evoked with short-term exposures during response operations. Depending on the goals of monitoring (immediate response, long-term recovery), communities may wish to additionally consider chemicals with health effects under long-term exposure scenarios.

### Step 5: designate locations for monitoring

The number of environmental monitoring locations may be limited due to cost constraints. In our work, we identified potential monitoring areas through estimation of possible environmental levels of fugitive chemicals. To carry out the analyses, we made a series of simplified calculations to estimate possible dispersion of fugitive chemicals from a source point and resulting chemical concentrations in an area. In the absence of measured concentrations and publicly available tools for sophisticated chemical dispersion modeling in urban floodwaters, our objective was to perform exposure assessments to predict possible fugitive chemical exposures for selected populations.

Using modeling techniques to estimate environmental concentrations of fugitive chemicals is beneficial in that poststorm monitoring can be focused (not exclusively) on neighborhood spots where high concentrations are expected and concentrations of one or more chemicals could reach dangerous levels. However, for collaborations that lack the ability to carry out intensive modeling applications, there are other criteria for selecting monitoring locations. Locations for monitoring can be selected based on proximity to sensitive populations (e.g., residences, schools, and daycare centers), proximity to chemical sources and vulnerability of chemical source point, or a combination of both. Publicly available data (e.g., records from Departments of Planning) may be used to identify sensitive population locations. For many communities, simply selecting areas or populations of concern may be adequate and justifiable for directing the locations of monitoring efforts.

### Step 6: develop a dissemination and communication plan

When it comes to environmental health risks, effective risk communication is critical to increasing awareness and behavior change.^30^ An assessment of who the relevant stakeholders are and what their informational needs are will increase the relevance of the dissemination plan. A priority stakeholder group for any community-based monitoring program is the residents of the community. Primary goals for dissemination to this group include education to build awareness of chemical risks and empowerment to protect themselves. Establishing a CSG can be a critical liaison between the collaborative and the broader community and can help with several objectives, including ensuring two-way interactions to encourage feedback, integrating the community in the development of messages and in the interpretation of results, and ensuring culturally and linguistically appropriate messaging and media outlets.^31^

Another group of stakeholders are those who have the ability to influence policy, including those in federal, state, and local government. To facilitate action, this group will need to know that any environmental monitoring data are valid and credible. Furthermore, it is important to have this group fully engaged in the monitoring plan from the start of the process. Informational briefs should communicate how important the issue is to residents and the future growth of the community. Front-line health professionals will also need to be aware of the potential chemical risks that exist in a community. Informational briefs that relate contamination-related health symptoms to chemical toxicities can be used to increase awareness among health professionals for signs and symptoms of chemical exposure in their patient population. Finally, community leaders, and representatives of large and small businesses need to be engaged in the issue and tapped as resources to develop solutions for better chemical security.

## Conclusion

Chemical exposure is often the “second insult” to communities ravaged by a weather disaster, with potentially more long-term implications than the initial direct damage. Environmental monitoring programs are needed to document the magnitude and scope of the problem and to understand where resources and interventions are most needed. However, once a disaster occurs, it is already too late to develop such a program. By following the six-step process we have outlined, communities can proactively develop an environmental monitoring program to illuminate the unknown consequences of fugitive chemical exposures and improve disaster preparedness, recovery, and response for vulnerable waterfront communities.

## Notes

^1^ Sheila Kaplan and Jack Healy. “Houston's Floodwaters Are Tainted, Testing Shows.” *The New York Times*, 11 September 2017.

^2^ K. Crossett, *et al.* “National Coastal Population Report, Population Trends from 1970 to 2020,” *National Oceanic and Atmospheric Administration, Department of Commerce, developed in partnership with the US Census Bureau.* <http:// coast. noaa. gov/ features/ population-report. pdf>. (Last accessed on December 17, 2017).

^3^ National Oceanic and Atmospheric Administration. U.S. Environmental Protection Agency, the International City/County Management Association, and Rhode Island Sea Grant. “Coastal & Waterfront Smart Growth” Available at https://coastalsmartgrowth.noaa.gov/report.html, Last accessed December 1, 2017.

^4^ Eddie Bautista, *et al.* “New York City Environmental Justice Alliance Waterfront Justice Project.” *Local Environment* 20 (2015).

^5^ P. Rutledge, *et al. Addressing Community Concerns: How Environmental Justice Relates to Land Use and Planning and Zoning*. (Washington, DC: National Academy of Public Administration, 2003).

^6^ See footnote 2.

^7^ Kerry Ard. “Trends in Exposure to Industrial Air Toxins for Different Racial and Socioeconomic Groups: A Spatial and Temporal Examination of Environmental Inequality in the US from 1995 to 2004.” *Social Science Research* 53 (2015).

^8^ Center for Science and Democracy at the Union of Concerned Scientists and Texas Environmental Justice Advocacy Services. “Double Jeopardy in Houston: Acute and Chronic Chemical Exposures Pose Disproportionate Risks for Marginalized Communities.” (2016). Available at: https://www.ucsusa.org/sites/default/files/attach/2016/10/ucs-double-jeopardy-in-houston-full-report-2016.pdf, Last accessed December 8, 2017.

^9^ Julie Sze. *Noxious New York: The Racial Politics of Urban Health and Environmental Justice* (MIT Press, 2006).

^10^ John Manuel. “In Katrina's Wake.” *Environmental Health Perspectives* 114 (2006).

^11^ Danny D. Reible, *et al. Toxic and Contaminant Concerns Generated by Hurricane Katrina*. (American Society of Civil Engineers, 2006).

^12^ Nicholas Santella, Laura J. Steinberg, and Hatice Sengul. “Petroleum and Hazardous Material Releases from Industrial Facilities Associated with Hurricane Katrina.” *Risk Analysis* 30 (2010).

^13^ Bautista, Eddie, *et al.* “New York City Environmental Justice Alliance Waterfront Justice Project.” *Local Environment* 20.6 (2015): 664–682.

^14^ Michael Bloomberg. “A Stronger, More Resilient New York.” *City of New York, PlaNYC Report* (2013).

^15^ Sandy Regional Assembly recovery agenda; Recovery from the ground up, strategies for community-based resiliency in New York and New Jersey. (2013). doi: https://doi.org/doi:10.7282/T3C24TN5.

^16^ Community Resiliency in Environmental Justice Industrial Waterfront Communities Work Group for the National Environmental Justice Advisory Council. “Proposed Recommendations for Promoting Community Resilience in Environmental Justice Industrial Waterfront Areas.” (2015). Available at https://www.epa.gov/sites/production/files/2016–08/documents/communityresilienceinejindustrialwaterfrontcommunities.pdf Last accessed on December 8, 2017.

^17^ Patrick L. Kinney, *et al.* “New York City Panel on Climate Change 2015 Reportchapter 5: Public Health Impacts and Resiliency.” *Annals of the New York Academy of Sciences* 1336 (2015).

^18^ Duane A. Gill and J. Steven Picou. “Technological Disaster and Chronic Community Stress.” *Society & Natural Resources* 11 (1998).

^19^ See footnote 1.

^20^ Robert D. Bullard and Glenn S. Johnson. “Environmentalism and Public Policy: Environmental Justice: Grassroots Activism and Its Impact on Public Policy Decision Making.” *Journal of Social Issues* 56 (2000).

^21^ Christopher Bacon, *et al.* “Introduction to Empowered Partnerships: Community-Based Participatory Action Research for Environmental Justice.” *Environmental Justice* 6 (2013).

^22^ Ibid.

^23^ Peggy M. Shepard. “Advancing Environmental Justice through Community-Based Participatory Research.” *Environmental Health Perspectives* 110, no. Suppl 2 (2002).

^24^ Liam R. O'Fallon and Allen Dearry. “Community-Based Participatory Research as a Tool to Advance Environmental Health Sciences,” *Environmental Health Perspectives* 110, no. Suppl 2 (2002).

^25^ See footnote 1.

^26^ Reible D.D., Haas C.N., Pardue J.H., Walsh W.J. (2006) Toxic and Contaminant Concerns Generated by Hurricane Katrina. *The Bridge* Vol 36 No.1 pp. 5–13.

^27^ Lynne Peeples. “Hurricane Sandy: Toxic Pollution, Low-Income Families in Direct Path of Storm Surges.” <https://www.huffingtonpost.com/2012/11/06/hurricane-sandy-pollution-low-income-families-storm-surge_n_2080241.html>. Last accessed December 8, 2017.

^28^ *See* footnote 13.

^29^ Environmental Protection Agency Office of the Administrator. “Status of Superfund Sites in Areas Affected by Harvey.” *News Release*, 2 September 2017, <https://www.epa.gov/newsreleases/status-superfund-sites-areas-affected-harvey>. Last accessed December 8, 2017.

^30^ Donna Fitzpatrick-Lewis, *et al.* “Communication About Environmental Health Risks: A Systematic Review.” *Environmental Health* 9 (2010).

^31^ Ibid.

